# Virulence genes identification in *Salmonella enterica* isolates from humans, crocodiles, and poultry farms from two regions in Colombia

**DOI:** 10.14202/vetworld.2023.2096-2103

**Published:** 2023-10-14

**Authors:** Julieth Michel Petano-Duque, Valentina Rueda-García, Iang Schroniltgen Rondón-Barragán

**Affiliations:** 1 Poultry Research Group, Faculty of Veterinary Medicine and Zootechnics, University of Tolima, Santa Helena Highs, Ibagué, Tolima, Colombia; 2 Research Group in Immunobiology and Pathogenesis, Faculty of Veterinary Medicine and Zootechnics, University of Tolima, Santa Helena Highs, Ibagué, Tolima, Colombia

**Keywords:** broilers cloaca, carcasses, crocodiles, gastroenteritis human, polymerase chain reaction, virulence genes

## Abstract

**Background and Aim:**

*Salmonella* spp. is frequently found in the digestive tract of birds and reptiles and transmitted to humans through food. Salmonellosis is a public health problem because of pathogenicity variability in strains for virulence factors. This study aimed to identify the virulence genes in *Salmonella* isolates from humans, crocodiles, broiler cloacas, and broiler carcasses from two departments of Colombia.

**Materials and Methods:**

This study was conducted on 31 *Salmonella enterica* strains from humans with gastroenteritis (seven), crocodiles (seven), broiler cloacas (six), and broiler carcasses (12) from Tolima and Santander departments of Colombia, belonging to 21 serotypes. All samples were tested for *Salmonella* spp. using culture method on selective and non-selective mediums. Extraction of genomic DNA was performed from fresh colonies, DNA quality was verified by spectrophotometry and confirmed by amplification of *InvA* gene using conventional polymerase chain reaction (PCR). *bapA*, *fimA*, *icmF*, *IroB*, *marT*, *mgtC*, *nlpI*, *oafA*, *pagN*, *siiD*, *spvC*, *spvR*, *spvB*, *Stn*, and *vexA* genes were amplified by PCR.

**Results:**

The most prevalent gene was *bapA* (100%), followed by *marT* (96.77%), *mgtC* (93.55%), and *fimA* (83.87%). Likewise, *IroB* (70.97%), *Stn* (67.74%), *spvR* (61.29%), *pagN* (54.84%), *icmF* (54.8%), and *SiiD* (45.16%) were positive for more than 50% of the strains. Furthermore, none of the isolates tested positive for the *vexA* gene. *Salmonella* isolates presented 26 virulence profiles.

**Conclusion:**

This study reported 14 virulence genes in *Salmonella* spp. isolates from humans with gastroenteritis, crocodiles, and broiler cloacas and carcasses. The distribution of virulence genes differed among sources. This study could help in decision-making by health and sanitary authorities.

## Introduction

*Salmonella* is a genus of Gram-negative bacteria from the *Enterobacteriaceae* family, classified into two species: *Salmonella* *bongori* and *Salmonella enterica*, commonly found in the digestive tract of mammals, birds, and reptiles. It represents a contagion source for humans through the consumption of foods such as beef, chicken meat, eggs, fish, pork, and vegetables [1–4]. *Salmonella enterica* has 2700 serotypes and subspecies that cause 99% of infections, of which 20 serotypes are zoonotic, including *Salmonella* Enteritidis, *Salmonella* Typhimurium, and *Salmonella* Heidelberg, the most relevant serotypes in public health [4–7].

*Salmonella* spp. are the etiological agents of several diseases, such as gastroenteritis, typhoid, paratyphoid, septicemia, and meningitis [2, 8, 9]. This zoonotic pathogen represents a public health problem leading per year to 93.8 million cases and 155,000 deaths worldwide; 1.35 million infections, 26,500 hospitalizations, and 420 deaths in the USA result in an estimated $400 million in direct medical costs, 70%–80% of food poisoning incidents in China, and every 690 out of 100,000 Europe inhabitants have non-typhoidal salmonellosis [2, 10, 11]. Moreover, a negative economic impact of $110 billion/per year has been estimated on the poultry industry [7, 12].

Serotype, inoculum amount, host immunological status, and virulence factors that influence strain pathogenicity are the main problems associated with salmonellosis prevention [7]. Moreover, genes on chromosomes, *Salmonella* pathogenicity islands (SPIs), mobile genetic elements (i.e., transposons, plasmids, and bacteriophages), and pili [12]; that code for adaptation to the host cell, resistance to antimicrobials, and the ability to overcome host defense mechanisms encoded virulence factors [7, 12]. Preliminary studies have established the presence and resistance to antibiotics of different serotypes of *S*. *enterica* in the poultry industry in Tolima and Santander departments, as well as in various isolates from humans and crocodiles (*Caiman crocodilus*) in the department of Tolima [9, 13–16]. To improve the understanding of the virulence profile and establish strategies that contribute to the control and prevention of salmonellosis, this study aimed to identify virulence genes in *S. enterica* isolates from patients with gastroenteritis, chicken carcasses and cloacal swabs, and crocodiles from two regions in Colombia.

## Materials and Methods

### Ethical approval

Ethical approval was not required for this study. *Salmonella* spp. strains were obtained from the bacterial strain collection of the Laboratory of Immunology and Molecular Biology (Universidad del Tolima).

### Study period and location

Strains were collected and isolated from March 2018 to November of 2022 in the Department of Tolima and Santander.

### Bacterial strains

Thirty-one strains of *S*. *enterica*, previously serotyped with the Kaffmann-White scheme, belonging to the serotypes *Salmonella* Braenderup, *Salmonella* Bovismorbificans, *Salmonella* Budapest, *S*. Enteritidis, *Salmonella* Grupensis, *S*. Heidelberg, *Salmonella* Hvittingofoss, *Salmonella* Infantis, *Salmonella* Javiana, *Salmonella* Kalina, *Salmonella* Manhattan, *Salmonella* Newport, *Salmonella* Othmarschen, *Salmonella* Paratyphi B, *Salmonella* Powell, *Salmonella* Saintpaul, *Salmonella* Schwarzengrund, *Salmonella* Skansen, *Salmonella* Soerenga, *S*. Typhimurium, and *Salmonella* Uganda were used. These isolates were otbained from human patients with gastroenteritis (n = 6), *Caiman crocodilus fuscus* (n = 7), and chicken carcasses (n = 12) from the department of Tolima, as well as from broiler cloacas from two regions in Colombia (Tolima, n = 3; Santander, n = 3) stored in the strain collection of the Laboratory of Immunology and Molecular Biology in the University of Tolima [16–19].

### Genomic DNA (gDNA) extraction

Frozen *Salmonella* colonies were thawed and seeded in Trypticase Soy Agar and Xylose Lysine Tergitol 4 medium (Oxoid, Germany). Genomic DNA was extracted from fresh colonies using the Wizard® gDNA Purification (Promega, USA) according to the manufacturer’s conditions.

### Polymerase chain reaction (PCR)

Molecular confirmation of the *S*. *enterica* strains was realized through amplification of a 282 bp fragment of the *InvA* gene by PCR (Table-1) [16, 17, 19–21]. Sixteen virulence genes were assessed in 31 isolates of *S*. *enterica* by amplification of each gene using specific primers described in Table-2 [4, 12, 22, 23]. Each reaction with a final volume of 25 μL was made up of 15.875 µL deionized distilled water, 5 μL of Flexi Buffer 5× Colorless GoTaq®, 1 µL of dNTPs, 1 µL of each primer (10 pmol/µL), 0.125 µL of GoTaq® Flexi DNA Taq polymerase (Promega), and as template 1 µL of gDNA. For all experiments, *S*. *enterica* ATCC 13076® strain was used as a reference strain and *Escherichia coli* strain as a negative control.

**Table-1:** Strains of *Salmonella enterica*.

Origin	Serotype	Code	Reference
Crocodiles	Saintpaul	LIBM0055	[16]
	Braenderup	LIBM0060	
	Soerenga	LIBM0061	
	Infantis	LIBM0063	
	Javiana	LIBM0066	
	Paratyphi B	LIBM0067	
	Powell	LIBM0068	
Broiler cloacas	Heidelberg	LIBM0011	[17]
	Heidelberg	LIBM0013	
	Heidelberg	LIBM0015	
	Paratyphi B	LIBM0017	[19]
	Paratyphi B	LIBM0022	
	Paratyphi B	LIBM0023	
Gastroenteritis in humans	Newport	LIBM0040	[20]
	Enteritidis	LIBM0041	
	Braenderup	LIBM0044	
	Uganda	LIBM0045	
	Typhimurium	LIBM0047	
	Grupensis	LIBM0048	
Carcasses	Newport	UT-SN14001	[21]
	Skansen	UT-SN14002	
	Kalina	UT-SN14003	
	Schwarzengrund	UT-SN14004	
	Paratyphi B	UT-SN14010	
	Manhattan	UT-SN14012	
	Braenderup	UT-SN14014	
	Bovismorbificans	UT-SN14016	
	Typhimurium	UT-SN14017	
	Othmarschen	UT-SN14019	
	Hvittingfoss	UT-SN14023	
	Budapest	UT-SN14045	

**Table-2:** Primers sequences and features.

Gene	Primer sequence	%GC	Tm (°C)	Amplicon size (bp)	Reference
*bapA*	F: TAAGCGTCGGACTTGGAATG	50.0	58	543	[4]
	R: CGTTCTTCAGCGTGTAGGTATAG	47.8	58.7		
*fimA*	F: CCTTTCTCCATCGTCCTGAA	50.0	56.9	85	[12]
	R: TGGTGTTATCTGCCTGACCA	50.0	58.6		
*icmF*	F: GCGTAGTCCAGATGAGACATTAG	47.8	58.5	724	[4]
	R: GCGGCCAGATAGACGATATTT	47.6	58		
*IroB*	F: TGCGTATTCTGTTTGTCGGTCC	61.1	50	606	[22]
	R: TACGTTCCCACCATTCTTCCC	59.7	52.4		
*marT*	F: CGTCGTCTCACAACAAACATTC	45.5	58.5	556	[4]
	R: CTGACAAATCAATGCCGTAACC	45.5	58.2		
*mgtC*	F: AAAGACAATGGCGTCAACGTATGG	45.8	62.2	500	[4]
	R: TTCTTTATAGCCCTGTTCCTGAGC	45.8	60.4		
*nlpl*	F: AGTCTTGGTTTGAGGGCATTAG	45.5	58.3	333	[4]
	R: TTCTTTCGCCTGCTTCTCATTA	40.9	58.1		
*oafA*	F: CGAGTGACTGGAACCAAAGA	50.0	57.5	510	[4]
	R: CAAGCATAGAGCCAGAGTAGAG	50.0	57.8		
*pagN*	F: TTCCAGCTTCCAGTACGTTTAG	45.5	58.1	440	[4]
	R: GCCTTTGTGTCTGCATCATAAG	45.5	57.9		
*siiD*	F: GTCAGGGCGTTATCACTACTAAA	43.5	58.0	826	[4]
	R: TTCACATCGGCCAGCATAG	52.6	57.6		
*spvC*	F: ACTCCTTGCACAACCAAATGCGGA	50.0	65.6	572	[12]
	R: TGTCTTCTGCATTTCGCCACCATCA	48.0	65.2		
*spvR*	F: CCGCTGAGCAGGGTTATTT	52.6	57.8	723	[4]
	R: CTTGGTCGGGTAATACAAGGAG	50.0	58.2		
*spvB*	F: CTATCAGCCCCGCACGGAGAGCAGTTTTTA	53.3	69.8	717	[4]
	R: GGAGGAGGCGGTGGCGGTGGCATCATA	66.7	73.5		
*Stn*	F: CTTTGGTCGTAAAATAAGGCG	44.4	52.4	260	[23]
	R: TGCCCAAAGCAGAGAGATTC	50.0	57.9		
*vexA*	F: AAACTAAGCGCTCCCGATAC	50.0	57.8	504	[4]
	R: CAGTCGCGCAGTGAAATAATG	47.6	58.3		

Polymerase chain reaction was performed in the ProFlex PCR System (Applied Biosystems, ThermoFisher, USA) following the parameters recommended by the manufacturer. The annealing temperature and extension time were defined based on the primer melting temperatures and the expected amplicon size. Products were detected by 2% agarose gel electrophoresis for 40 min at 100 V using PowerPac™ equipment (Bio-Rad, USA), HydraGreen™ as DNA dye (ACTGene, USA), and the ENDURO GDS gel documentation system (Labnet International, USA).

## Results

All 31 *S. enterica* strains were tested by PCR for virulence genes presence. The amplification of gene operon invasion A (*InvA*) confirmed the presence of *Salmonella* in nearly all strains (96.77%), except for the *Salmonella* Kalina strain isolated from chicken carcasses as shown in Table-3.

**Table-3:** Virulence gene patterns in *Salmonella enterica* strains.

Sources	Serotype	Virulence genes																Profiles

		SPI-1			SPI-2	SPI-3		SPI-4	SPI-6	SPI-7	SPI-8	SPI-9	SPI-19	Chromosomal	Plasmids			
									
		*fimA*	*InvA*	*IroB*	*oafA*	*marT*	*mgtC*	*siiD*	*pagN*	*vexA*	*nlpI*	*bapA*	*icmF*	*Stn*	*spvR*	*spvB*	*spvC*	
Crocodiles	Braenderup	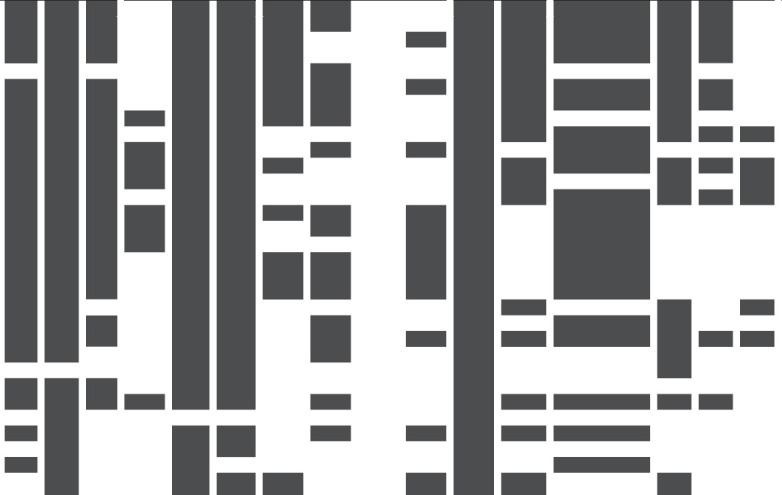																I
	Infantis																	I
	Javiana																	II
	Paratyphi B																	III
	Powell																	IV
	Saintpaul																	II
	Soerenga																	I
Gastroenteritis in humans	Braenderup																	V
	Enteritidis																	VI
	Grupensis																	VII
	Newport																	VIII
	Typhimurium																	IX
	Uganda																	X
Broiler cloacas	Heidelberg																	XI
	Heidelberg																	XII
	Heidelberg																	XIII
	Paratyphi B																	XIV
	Paratyphi B																	XIV
	Paratyphi B																	XIV
Carcasses	Bovismorbificans																	XV
	Braenderup																	XVI
	Budapest																	XVII
	Hvittingfoss																	XVIII
	Kalina																	XIX
	Manhattan																	XX
	Newport																	XXI
	Othmarschen																	XXII
	Paratyphi B																	XXIII
	Schwarzengrund																	XXIV
	Skansen																	XXV
	Typhimurium																	XXVI

**Figure-1:**
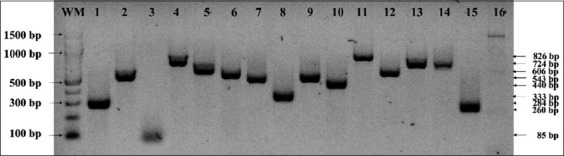
Representative amplification of virulence genes from *Salmonella enterica* isolates. 1. *InvA* gene amplicon (284 bp); 2. *bapA* gene amplicon (543 bp); 3. *fimA* gene amplicon (85 bp); 4. *icmF* gene amplicon (724 bp); 5. *IroB* gene amplicon (606 bp); 6. *marT* gene amplicon (556 bp); 7. *mgtC* gene amplicon (500 bp); 8. *nlpl* gene amplicon (333 bp); 9. *oafA* gene amplicon (510 bp); 10. *pagN* gene amplicon (440 bp); 11. *siiD* gene amplicon (826 bp); 12. *spvC* gene amplicon (572 bp); 13. *spvR* gene amplicon (723 bp); 14. *ssvB* gene amplicon (717 bp); 15. *Stn* gene amplicon (260 bp); 16. *vexA* gene amplicon (504 bp). WM: Weight marker 100 bp (New England, Biolabs, USA). Agarose gel 2%.

In this study, 12 virulence genes present in eight SPIs, one gene on chromosomal, and three genes of plasmids were evaluated. Regarding virulence genes frequencies, among different serovars denoting variable rates, the most prevalent gene was biofilm-associated protein A (*bapA;* 100%), followed by *marT* (96.77%), *mgtC* (93.55%), and Type IV fimbrial subunit (*fimA*; 83.87%) (Figure-1 and Table-3). Likewise, *IroB* (70.97%), enterotoxin (*Stn*; 67.74%), *Salmonella* virulence plasmid R (*spvR*; 61.29%), phoP-activated gene (*pagN*; 54.84%), intracellular multiplication protein F (*icmF*; 54.8%), and *SiiD* (45.16%) were positive for more than 50% of the strains (Figure-1 and Table-3). In addition, *nlpI* (38.71%), *Salmonella* virulence plasmid B (*spvB*; 35.48%), O-acetyltransferase (*oafA*; 25.81%), and *Salmonella* virulence plasmid C (*spvC*; 19.35%) genes were in a lower frequency (Figure-1, Table-3). Furthermore, none of the isolates tested positive for the *vexA* gene. Overall, 19 strains had more than nine virulence genes isolates from all gastroenteritis cases in humans, including *Paratyphi B* and Newport *Salmonella* strains (Table-3).

The distribution of genes according to strain origin showed that 100% of the crocodiles, gastroenteritis, and cloacas presented *marT* and *mgtC* genes (Table-3). Furthermore, 100% of gastroenteritis in humans and cloacas in broiler isolates carried *fimA* and *iroB* gene (Table-3). Moreover, *icmF*, *siiD*, and *spvR* were present in 100% crocodile strains; and the *nlpI* gene present in 100% cloaca strains (Table-3). Neither crocodile nor cloaca chicken isolates had *spvC* (Table-3). In addition, no chicken cloaca strains presented *icmF*, *spvB*, and *Stn* genes, and *oafA* was not present in the crocodile strains (Table-3).

In addition, the virulence genes presence was classified into 26 profiles (P) (Table-3). Crocodile isolates had four genetic profiles (PI–PIV), six patrons in cases of human gastroenteritis (PV–PX), four profiles (PXI–PXIV) from broiler cloacas, and 12 profiles in broiler carcasses (PXV–PXXVI) (Table-3). PVII and PXVII were the genetic profiles with more virulence genes present (13 genes), followed by PI, PII, and PXXI with 12 genes, and PXXII had 2 genes being the patron with less virulence genes (Table-3). Most of the profiles were found once, except for PI present in the isolates of *S*. Braenderup, *S*. Infantis, and *S*. Soerenga from crocodiles, the PII found in *S*. Javiana and *S*. Saintpaul from crocodiles, and the PXIV detected in the three strains of *S*. Paratyphi B from broiler cloacas (Table-3).

## Discussion

Virulence genes of *Salmonella* isolates are in 23 SPI, chromosomal, and plasmids [24]. The data in this study showed that 14 virulence genes were detected, but their distribution differed among source and not among serotypes, as other studies reported [4, 19, 25]. Virulence genes of SPI-1 to SPI-5 are common in all *Salmonella* isolates [24]. Accordingly, *fimA* (SPI-1), *iroB* (SPI-1), *marT* (SPI-3), *mgtC* (SPI-3), and *siiD* (SPI-4) genes were found in *Salmonella* strains frequently.

*Salmonella* pathogenicity islands-1 and SPI-2 possess many virulence genes associated with extracellular pathogenesis and co-encode Type III secretion system [24]. In this regard, the genes located in SPI-1 have been described and characterized in *S*. Typhimurium strains [26]. However, *S*. Typhimurium isolates from broiler carcasses did not have *fimA* and *iroB* genes of SPI-1 (Table-3). Other strains did not present these genes, such as *S*. Powel, *S*. Othmarschen, *S*. Schwarzengrund, and *S*. Typhimurium (broilers carcasses) (Table-3). *fimA* gene is necessary for the aggregation of Type I fimbriae; in turn, the symbioses are essential for the colonization and biofilm formation of *Salmonella* spp. [27, 28]. Furthermore, the *iroB* gene encodes the glycosyltransferase that glycosylates enterobactin, preventing the host antimicrobial protein from sequestering the iroBCDEN siderophore [29–31].

*Salmonella* pathogenicity islands-2, SPI-3, and SPI-6-8 contain genes that allow *Salmonella* isolates to resist acidic environments, replicate intracellularly, and escape the host’s immune system [24]. According to the roles played by SPI-2 effector genes, the presence of *oafA* gene in humans with gastroenteritis and broiler cloacas could be due to the use of the acetylation reaction in cell infection, leading to the increased antimicrobial activity of macrophage and cell growth [4, 24, 32, 33].

Regarding SPI-3, *marT* and *mgtC* genes were present in most strains (Table-3), which agrees with previous reports by Yue *et al*. [4]. *marT* gene causes systemic infection because it plays a significant role in metabolism within the phagosome and may act as a general pathogenicity regulator by overexpression genes encoding main proteins in the fimbriae formation (e.g., *fimA* gene), biofilm regulators (e.g., *nlpI* gene), large surface proteins, antigenic surface proteins, and flagellar operons [34, 35]. The *marT* gene absence coincided with the *fimA* and *nlpI* genes lack in *S*. Othmarschen strain (Table-3). Besides, the *mgtC* gene is linked with independent flagellar growth and motility at low concentrations of Mg^+2^ [36]. According to this, *S*. Othmarschen and *S*. Skansen of broiler carcasses were not lacked Mg^+2^ (Table-3). Moreover, the *mgtC* gene encodes the binding protein MgtC that plays a regulatory role in complex mgtCBR and mediates phosphate transport necessary for *Salmonella* spp. pathogenesis [37, 38].

*siiD* gene of SPI-4 was found in *S*. Paratyphi B isolates from crocodiles and broiler cloacas but was absent in the strain from broiler carcasses, as described by Yue *et al*. [4]. Likewise, it has a low occurrence in *S*. Enteritidis and *S*. Typhimurium strains [4], according to gene expression in one of the two *S*. Typhimurium strains and the absence in *S*. Enteritidis (Table-3). The fact that the strains have this gene denotes the union of the inner and outer membranes with the putative membrane fusion protein, a component of the Type I secretion system [39].

*Salmonella* pathogenicity islands-6 encodes the Type 6 secretion system that leads to survival within macrophages and successful establishment in the host intestine [40, 41]. *pagN* gene confers competitive advantages to the strains because it promotes hemagglutination, contributing to the adhesion of the pathogen to mammalian cells [42]. Furthermore, the *pagN* gene is related to acidified environments, low Mg^+2^ concentrations, or the presence of antimicrobial peptides [43]. In this way, it is possible to suggest that the *S*. Othmarschen and *S*. Skansen strains were in environments with a low concentration of Mg^+2^ because these isolates did not have the *mgtC* gene either (Table-3).

*vexA* gene is involved in the biosynthesis and export of capsule VI to the cell surface [44]. This gene was not found in the serotypes from crocodiles, human cases of gastroenteritis, and poultry farms (Table-3), and other studies reported its absence in *S*. Typhimurium and *Salmonella* Dublin [24, 45]. On the other hand, *nlpI* gene is linked to biofilm formation and acclimation of *S*. Typhimurium [46, 47], even though strain from human gastroenteritis lacked this gene.

*bapA* gene was present in all serotypes (Table-3), since codes for a large-secreted protein required for biofilm formation and host colonization [48]. In the case of the *icmF* gene, it encodes for an inner membrane protein of Type 6 system secretion that contributes to the virulence of *Salmonella* spp. [24, 49].

On the other hand, the *stn* gene chromosomal operon induces a loss of intestinal fluids, causing diarrhea and leading to severe acute gastroenteritis [50]. According to this, the *stn* gene was present in 4/6 serotypes from human gastroenteritis cases (Table-3). Furthermore, the *Stn* gene may affect membrane integrity of *Salmonella* spp. through ompA localization regulation [51].

*Salmonella* virulence plasmid operon (spvRABCD) expression is induced by the host cells’ intracellular environment, and operon genes are involved in survival and intracellular growth, and macrophage killing [24, 52]. The isolates that presented three plasmid genes (*spvR*, *spvB*, and *spvC*) include *S*. Budapest *S*. Enteritidis, *S*. Newport (gastroenteritis in humans), and *S*. Uganda. *spvR* and *spvBC* genes are required for the virulence phenotype of the *spv* operon [52]. *spvB* gene was found in *S*. Braenderup, *S*. Enteritidis, *S*. Infantis, *S*. Javiana, *S*. Paratyphi B, *S*. Saintpaul, *S*. Soerenga, and *S*. Uganda, as well as in the *S*. Newport strains of cases of gastroenteritis and carcasses in broiler chickens. Nevertheless, Yue *et al*. [4] reported *spvB* gene in *S*. Typhimurium.

The presence of the *spvC* gene may be related to evading MAPK signaling, suppressing the inflammatory response, and spreading the bacteria in the late stages in specific serotypes [24, 53, 54]. This agrees with its presence in two *S*. Typhimurium isolates from different sources (human gastroenteritis and broiler carcasses). Similarly, the prevalence of the *spvC* gene is higher in *S*. Typhimurium and *S*. Enteritidis [55], which is consistent with its finding in both serotypes.

## Conclusion

This study reported 14 virulence genes in *Salmonella* spp. isolates from humans with gastroenteritis, crocodiles, and broiler cloacas, and broiler carcasses. The distribution of virulence genes differed among sources. Our results contribute to the characterization and monitoring of *S. enterica* isolates and their evolutionary process in the host from two departments of Colombia, and it could help in decision-making by health and sanitary authorities.

## Authors’ Contributions

JMPD and ISRB: Study conception and design and drafted the manuscript. JMPD and VRG: Conducted the experiments and analyzed the data. ISRB, VRG, and JMPD: Revised the manuscript. All authors have read, reviewed, and approved the final manuscript.

## References

[1] Dib A.L, Agabou A, Chahed A, Kurekci C, Moreno E, Espigares M, Espigares E (2018). Isolation, molecular characterization and antimicrobial resistance of *Enterobacteriaceae* isolated from fish and seafood. Food Control.

[2] Xie T, Wu G, He X, Lai Z, Zhang H, Zhao J (2019). Antimicrobial resistance and genetic diversity of *Salmonella enterica* from eggs. Food Sci. Nutr.

[3] Yang X, Wu Q, Huang J, Wu S, Zhang J, Chen L, Wei X, Ye Y, Li Y, Wang J, Lei T, Xue L, Pang R, Zhang Y (2020). Prevalence and characterization of *Salmonella* isolated from raw vegetables in China. Food Control.

[4] Yue M, Li X, Liu D, Hu X (2020). Serotypes, antibiotic resistance, and virulence genes of *Salmonella* in children with diarrhea. J. Clin. Lab. Anal.

[5] Jajere S.M (2019). A review of *Salmonella enterica* with particular focus on the pathogenicity and virulence factors, host specificity and antimicrobial resistance including multidrug resistance. Vet. World.

[6] Rady M, Ezz-El-Din N.A, Mohamed K.F, Nasef S, Samir A, Elfeil W.K (2020). Correlation between EsbL *Salmonella* serovars isolated from broilers and their virulence genes. J. Hellenic Vet. Med. Soc.

[7] Webber B, Borges K.A, Furian T.Q, Rizzo N.N, Tondo E.C, Dos Santos L.R, Rodrigues L.B, do Nascimento V.P (2019). Detection of virulence genes in *Salmonella Heidelberg* isolated from chicken carcasses. Rev. Inst. Med. Trop. Sao Paulo.

[8] Castro-Vargas R.E, Herrera-Sánchez M.P, Rodríguez-Hernández R, Rondón-Barragán I.S (2020). Antibiotic resistance in *Salmonella* spp. isolated from poultry:A global overview. Vet. World.

[9] Herrera-Sánchez M.P, Castro-Vargas R.E, Fandiño-de-Rubio L.C, Rodríguez-Hernández R, Rondón-Barragán I.S (2021). Molecular identification of fluoroquinolone resistance in *salmonella* spp. isolated from broiler farms and human samples obtained from two regions in Colombia. Vet. World.

[10] Balasubramanian R, Im J, Lee J.S, Jeon H.J, Mogeni O.D, Kim J.H, Rakotozandrindrainy R, Baker S, Marks F (2019). The global burden and epidemiology of invasive non-typhoidal *Salmonella* infections. Hum. Vaccin. Immunother.

[11] Centers for Disease Control and Prevention (2019). Antibiotic Resistance Threats in the United States.

[12] Nikiema M.E.M, Kakou-Ngazoa S, Ky/Ba A, Sylla A, Bako E, Addablah A.Y.A, Ouoba J.B, Sampo E, Gnada K, Zongo O, Traoré K.A, Sanou A, Bonkoungou I.J.O, Ouédraogo R, Barro N, Sangaré L (2021). Characterization of virulence factors of *Salmonella* isolated from human stools and street food in urban areas of Burkina Faso. BMC Microbiol.

[13] Cruz-Méndez J.S, Ortiz-Muñoz J.D, Rondón-Barragán I.S (2022). Genotyping of *Salmonella enterica* strains from animal and human origin using three molecular techniques. Iraqi J. Vet. Sci.

[14] Herrera-Sánchez M.P, Rodríguez-Hernández R, Rondón-Barragán I.S (2020). Molecular characterization of antimicrobial resistance and enterobacterial repetitive intergenic consensus-PCR as a molecular typing tool for *Salmonella* spp. isolated from poultry and humans. Vet. World.

[15] Rodríguez-Hernández R, Herrera-Sánchez M.P, Ortiz-Muñoz J.D, Mora-Rivera C, Rondón-Barragán I.S (2022). Molecular characterization of *Salmonella* spp. isolates from Wild Colombian Babilla (*Caiman crocodilus fuscus*) isolated *in situ*. Animals (Basel).

[16] Lozano-Villegas K, Rodríguez-Hernández R, Rondón-Barragán I (2019). Effectiveness of six molecular typing methods as epidemiological tools for the study of *Salmonella* isolates in two Colombian regions. Vet. World.

[17] Castro-Vargas R, de Rubio L.C.F, Vega A, Rondon-Barragan I (2019). Phenotypic and genotypic resistance of *Salmonella Heidelberg* isolated from one of the largest poultry production regions from Colombia. Int. J. Poult. Sci.

[18] Rodríguez R, Fandiño C, Donado P, Guzmán L, Verjan N (2015). Characterization of *Salmonella* from commercial egg-laying hen farms in a central region of Colombia. Avian Dis.

[19] Rodríguez-Hernández R, Bernal J.F, Cifuentes J.F, Fandiño L.C, Herrera-Sánchez M.P, Rondón-Barragán I, Garcia N.V (2021). Prevalence and molecular characterization of *salmonella* isolated from broiler farms at the Tolima region-Colombia. Animals (Basel).

[20] Fandiño L.C, Verjan N (2019). A common *Salmonella enteritidis* sequence type from poultry and human gastroenteritis in Ibagué, Colombia. Un tipo de secuencia común de *Salmonella enteritidis* de origen aviar y de humano con gastroenteritis en Ibagué, Colombia. Biomedica.

[21] Vélez D.C, Rodríguez V, García N.V (2017). Phenotypic and genotypic antibiotic resistance of *Salmonella* from chicken carcasses marketed at Ibague, Colombia. Braz. J. Poult. Sci.

[22] Soubeiga A.P, Kpoda D.S, Compaoré M.K.A, Somda-Belemlougri A, Kaseko N, Rouamba S.S, Ouedraogo S, Traoré R, Karfo P, Nezien D, Nikiéma F, Kabre E, Zongo C, Savadogo A (2022). Molecular characterization and the antimicrobial resistance profile of *Salmonella* spp. isolated from ready-to-eat foods in Ouagadougou, Burkina Faso. Int. J. Microbiol.

[23] Wójcicki M, Chmielarczyk A, Świder O, Średnicka P, Strus M, Kasperski T, Shymialevich D, Cieślak H, Emanowicz P, Kowalczyk M, Sokołowska B, Juszczuk-Kubiak E (2022). Bacterial pathogens in the food industry:Antibiotic resistance and virulence factors of *Salmonella enterica* strains isolated from food chain links. Pathogens.

[24] Wang M, Qazi I.H, Wang L, Zhou G, Han H (2020). *Salmonella* virulence and immune escape. Microorganisms.

[25] Lozano-Villegas K.J, Herrera-Sánchez M.P, Beltrán-Martínez M.A, Cárdenas-Moscoso S, Rondón-Barragán I.S (2023). Molecular detection of virulence factors in *Salmonella serovars* isolated from poultry and human samples. Vet. Med. Int.

[26] Johnson R, Mylona E, Frankel G (2018). Typhoidal *Salmonella*:Distinctive virulence factors and pathogenesis. Cell. Microbiol.

[27] Koo H, Allan R.N, Howlin R.P, Stoodley P, Hall-Stoodley L (2017). Targeting microbial biofilms:Current and prospective therapeutic strategies. Nat. Rev. Microbiol.

[28] Meng X, Meng X, Wang J, Wang H, Zhu C, Ni J, Zhu G (2019). Small non-coding RNA STnc640 regulates expression of *fimA* fimbrial gene and virulence of *Salmonella enterica* serovar Enteritidis. BMC Vet. Res.

[29] Hantke K, Nicholson G, Rabsch W, Winkelmann G (2003). Salmochelins, siderophores of *Salmonella enterica* and uropathogenic *Escherichia coli* strains, are recognized by the outer membrane receptor IroN. Proc. Natl. Acad. Sci. U. S. A.

[30] Fischbach M.A, Lin H, Zhou L, Yu Y, Abergel R.J, Liu D.R, Smith K.D (2006). The pathogen-associated *iroA* gene cluster mediates bacterial evasion of lipocalin 2. Proc. Natl. Acad. Sci. U. S. A.

[31] Raffatellu M, George M.D, Akiyama Y, Hornsby M.J, Nuccio S.P, Paixao T.A, Bäumler A.J (2009). Lipocalin-2 resistance confers an advantage to *Salmonella enterica* serotype Typhimurium for growth and survival in the inflamed intestine. Cell Host Microbe.

[32] Guiney D.G, Fierer J (2011). The role of the *spv* genes in *Salmonella* pathogenesis. Front. Microbiol.

[33] Hauser E, Junker E, Helmuth R, Malorny B (2011). Different mutations in the *oafA* gene lead to loss of O5-antigen expression in *Salmonella enterica* serovar Typhimurium. J. Appl. Microbiol.

[34] Bertelloni F, Tosi G, Massi P, Fiorentini L, Parigi M, Cerri D, Ebani V.V (2017). Some pathogenic characters of paratyphoid *Salmonella enterica* strains isolated from poultry. Asian Pac. J. Trop. Med.

[35] Eran Z, Akçelik M, Yazıcı B.C, Özcengiz G, Akçelik N (2020). Regulation of biofilm formation by marT in *Salmonella* Typhimurium. Mol. Biol. Rep.

[36] Park S.Y, Pontes M.H, Groisman E.A (2015). Flagella-independent surface motility in *Salmonella enterica* serovar Typhimurium. Proc. Natl. Acad. Sci. U. S. A.

[37] Lee J.W, Lee E.J (2015). Regulation and function of the *Salmonella* MgtC virulence protein. J. Microbiol.

[38] Choi S, Choi E, Cho Y.J, Nam D, Lee J, Lee E.J (2019). The *Salmonella* virulence protein MgtC promotes phosphate uptake inside macrophages. Nat. Commun.

[39] Morgan E, Bowen A.J, Carnell S.C, Wallis T.S, Stevens M.P (2007). SiiE is secreted by the *Salmonella enterica* serovar Typhimurium pathogenicity island 4-encoded secretion system and contributes to intestinal colonization in cattle. Infect. Immun.

[40] Mulder D.T, Cooper C.A, Coombes B.K (2012). Type VI secretion system-associated gene clusters contribute to pathogenesis of *Salmonella enterica* serovar Typhimurium. Infect Immun.

[41] Sana T.G, Flaugnatti N, Lugo K.A, Lam L.H, Jacobson A, Baylot V, Monack D.M (2016). *Salmonella* Typhimurium utilizes a T6SS-mediated antibacterial weapon to establish in the host gut. Proc. Natl. Acad. Sci. U. S. A.

[42] Lambert M.A, Smith S.G.J (2008). The PagN protein of *Salmonella enterica* serovar Typhimurium is an adhesin and invasin. BMC Microbiol.

[43] Barilleau E, Védrine M, Koczerka M, Burlaud-Gaillard J, Kempf F, Grépinet O, Wiedemann A (2021). Investigation of the invasion mechanism mediated by the outer membrane protein PagN of *Salmonella* Typhimurium. BMC Microbiol.

[44] Daigle F, Graham J.E, Curtiss R (2001). Identification of *Salmonella typhi* genes expressed within macrophages by selective capture of transcribed sequences (SCOTS). Mol. Microbiol.

[45] Mohammed M, Delappe N, O'connor J, McKeown P, Garvey P, Cormican M (2016). Whole genome sequencing provides an unambiguous link between *Salmonella Dublin* outbreak strain and a historical isolate. Epidemiol. Infect.

[46] Rouf S.F, Ahmad I, Anwar N, Vodnala S.K, Kader A, Romling U, Rhen M (2011). Opposing contributions of polynucleotide phosphorylase and the membrane protein NlpI to biofilm formation by *Salmonella enterica* serovar Typhimurium. J. Bacteriol.

[47] Rouf S.F, Anwar N, Clements M.O, Rhen M (2011). Genetic analysis of the pnp-deaD genetic region reveals membrane lipoprotein NlpI as an independent participant in cold acclimatization of *Salmonella enterica* serovar Typhimurium. FEMS Microbiol. Lett.

[48] Latasa C, Roux A, Toledo-Arana A, Ghigo J.M, Gamazo C, Penadés J.R, Lasa I (2005). BapA, a large secreted protein required for biofilm formation and host colonization of *Salmonella enterica* serovar Enteritidis. Mol. Microbiol.

[49] Ma L.S, Narberhaus F, Lai E.M (2012). IcmF family protein TssM exhibits ATPase activity and energizes Type VI secretion. J. Biol. Chem.

[50] ElSheikh M, Abdeen E, Ammar A (2019). Molecular detection of some virulence genes of *Salmonella* serotypes isolated from poultry in Egypt. J. Curr. Vet. Res.

[51] Nakano M, Yamasaki E, Ichinose A, Shimohata T, Takahashi A, Akada J.K, Kurazono H (2012). *Salmonella* enterotoxin (Stn) regulates membrane composition and integrity. Dis. Model Mech.

[52] Passaris I, Cambré A, Govers S.K, Aertsen A (2018). Bimodal expression of the *Salmonella Typhimurium* spv operon. Genetics.

[53] Fardsanei F, Dallal M.M.S, Salehi T.Z, Douraghi M, Memariani M, Memariani H (2021). Antimicrobial resistance patterns, virulence gene profiles, and genetic diversity of *Salmonella enterica* serotype Enteritidis isolated from patients with gastroenteritis in various Iranian cities. Iran. J. Basic Med. Sci.

[54] Long L, You L, Wang D, Wang M, Wang J, Bai G, Li J, Wei X, Li S (2022). Highly prevalent MDR, frequently carrying virulence genes and antimicrobial resistance genes in *Salmonella enterica* serovar 4,[5],12:i:- isolates from Guizhou Province, China. PLoS One.

[55] Proroga Y.T.R, Capuano F, Capparelli R, Bilei S, Bernardo M, Cocco M.P, Campagnuolo R, Pasquele V (2018). Characterization of non-typhoidal *Salmonella enterica* strains of human origin in central and southern Italy. Ital. J. Food Saf.

